# Ball bearing fault detection using an acoustic based machine learning approach

**DOI:** 10.1038/s41598-025-33978-5

**Published:** 2025-12-29

**Authors:** C. B. Chandrakala, Sai Sreevalli Karumanchi, G. p Raghudathesh, N. Madhwesh

**Affiliations:** 1 Manipal Institute of Technology, Manipal Academy of Higher Education, Manipal, 576104 Karnataka India; 2 Manipal School of Information Sciences, Manipal Academy of Higher Education, Manipal, 576104 Karnataka India; 3 Department of Mechanical and Industrial Engineering, Manipal Institute of Technology, Manipal Academy of Higher Education, Manipal, 576104 Karnataka India

**Keywords:** Acoustic signals, Fault detection, CNN, Random forest, XGBoost, Scalograms, LeNet, Engineering, Mathematics and computing

## Abstract

The effective operation of ball bearings is crucial for industrial machinery, transportation equipment, and any mobile entity. The unforeseen malfunction of these bearings can lead to costly downtime, as well as potential damage and maintenance requirements. Predictive maintenance systems utilizing machine learning (ML) and deep learning (DL) methodologies have emerged as a promising solution for the early detection of bearing failures. This work aims to tackle the issue of predicting ball bearing faults by employing acoustic vibration signals using continuous wavelet transform-based deep neural networks. The time-frequency behavior of vibration signals given by sensors placed at both the faulty fan-end and drive-end sections of the ball-bearing is examined simultaneously. This is achieved by transforming numerical signals into stacked scalograms using non-overlapping windows. Subsequently, a convolutional neural network is trained utilizing the LeNet-5 architecture to classify the operating condition of the bearing into distinct defective states. The proposed method employs a novel approach that combines wavelet-based scalograms with convolution networks to achieve real-time data classification for the purpose of fault detection in ball bearings. The model developed in this study demonstrates a notable improvement in accuracy compared to other contemporary machine learning models for fault diagnosis as reported in the existing literature. The proposed model has a higher prediction accuracy of 99.23% than the numerical signal-based classification models.

## Introduction

A ball bearing is a special rolling-element bearing with three primary functions: to position moving machine parts, support radial and axial loads, and, most importantly, reduce friction, thus giving it a significant role in rotating systems. Their functionalities are achieved by placing them between two “races” or bearing rings to reduce the surface contact, thereby reducing the friction when compared to flat surfaces rubbing against each other. The above feature improves the machine’s lifetime by reducing machine parts’ wear and tear and reducing friction-generated heat.

Ball bearings might fail when subjected to moisture, overheating, rapid movements, and improper lubrication. Additionally, improper installation due to loose fits can cause misalignment. Putting too much load on the bearing is another cause of premature bearing failures. Oversights like this cause catastrophic damage, leading to systemic failure. While specific faults can be visible to the naked eye, many must be precisely measured using various variables like the type of signal, vibration amplitude, and frequency. Furthermore, with the help of machine learning and AI, these faults can be detected at early stages, preventing safety hazards while saving time and money. Therefore, conditional monitoring of rotating machinery is of vital importance.

Many ways exist to detect a fault, but vibration monitoring is frequently employed to distinguish between a normal and a faulty bearing. A normally functioning bearing generates vibration, but the presence of defects amplifies the signal significantly. From this data, important signal features can be extracted in multiple ways. We follow two approaches: namely, the Mel spectrum and the wavelet transforms. Mel cepstrum is used in sound processing to investigate periodic structure in the frequency spectra of an audio signal using the Mel scale. In the Mel scale, the frequency bands are equally spaced so that the bands together approximate the human auditory system’s response more closely than the linearly spaced frequency bands used in any normal spectrum. The vibration signals in ball bearings are acoustic in nature; we first follow this approach to classify the signals into different faulty classes using two machine learning models, XGBoost and Random Forest, applied to MFCC coefficients. However, in the data corresponding to faulty bearings, sporadic, non-periodic, and short-length spurts in the signal are noticed. Because the Mel cepstrum is based on Fourier coefficients, it can represent only periodic signals effectively. So, it is hypothesised that MFCC can only model long enough local behaviours. To overcome this shortcoming, in our second model, we follow the time-frequency approach using the wavelet transform method and generate short-term time-frequency maps for window-size signals. A mother wavelet is considered, which is a short waveform that can expand or shrink with the help of transformations. Any local behaviour in signals, like short-term spikes, for example, can be approximated by applying a suitable transformation to the mother wavelet. Then, as in the case of MFCC coefficients, a scalogram gives the coefficients of different transformations needed to locally approximate the signal as the wavelet scans the entire signal with sliding windows. This unique advantage of wavelets in capturing the local behavior motivated us to use the scalograms for faulty signal detection. The sequence of scalogram images constructed from the full signal is fed to the LeNet5 deep learning network for fault classification. In the case of fault detection, multiple sensors are used to measure vibrations at different locations, like the fan-end and the drive end of bearings, in our case. Relationships or interactions among these measurements can be effectively captured only when they are analyzed simultaneously. Hence, in each short time window, the scalograms of all the signals (fan-end and drive-end signals, in our study) are captured simultaneously to create a stack of aligned scalograms corresponding to that time window. The sequence of such stacked scalograms generated is given, whereas the time window along the signal is given as the input to the deep learning network LeNet-5. The selection of LeNet over other advanced and deeper networks is mainly motivated by its convincing performance with a relatively shallow architecture and by the limited size of the training dataset.

Extensive research has been undertaken in the field of bearing health monitoring with the aim of predicting and detecting failures in bearings. This is due to the potential consequences of bearing faults, which can lead to system failure or damage to the overall machine. The methods used for diagnosing bearing faults can be categorised into two groups: statistical approaches and probabilistic methods^1,2^. The statistical methodology involves the quantification and analysis of physical data pertaining to bearing faults, including factors such as vibration, heat, sound, pressure, and other factors, and other relevant parameters. This is achieved through the utilization of diverse sensors to capture the data, followed by numerical analytic techniques to statistically diagnose and identify problems^3,4^. Probabilistic methods, like support vector machines (SVMs), random forests, and artificial neural networks (ANNs), employ the use of collected/synthesised physical data as training data^5–7^. The statistical method can be employed to obtain data on bearing faults from numerous factors such as vibration, heat, sound, pressure, and other relevant factors. M. Delgado et.al^8^ present a new scheme for detecting and classifying both localised and distributed faults in bearings based on statistical-time features, curvilinear component analysis. The methodology uses a double feature reduction stage to analyse the significance of a set of statistical time features and employs discriminant analysis to quantify the features in relation to the considered classes and working conditions which allows for feature selection to maximize the discrimination between different faults and can provide deep insights into the bearing condition and fault status. The proposed methodology has been validated and found to be effective and feasible in different operating conditions^9^. proposes a fault diagnosis method for rotating machinery using Infrared Thermal (IRT) images, using a Convolutional Neural Network (CNN) to extract fault features from the IRT images, and the obtained features are fed into the Softmax Regression (SR) classifier for fault pattern identification. IRT Images are obtained from rotating machinery while subjected to 10 different health situations. During this time, the impact of the temperature has also been analysed as part of this research. Results demonstrate its superiority over Deep Belief Network (DBN), Deep Neural Network (DNN), and Stacked Autoencoder (SAE) in identifying ten health conditions of rotating machinery. The study^10^ focuses on the design of mixture de-noising for detecting faulty bearing signals under actual plant conditions. Mixture de-noising, which combines an adaptive noise-cancelling filter and a wavelet-based de-noise estimator, is found to be effective in improving the fault diagnostics of bearings by improving the signal-to-noise ratio^11^. investigated different time domain features for the diagnosis of rolling element bearings using acoustic emission (AE) signals. The selected time domain features for diagnosis purposes are the clearance factor, sixth central moment, impulse factor, crest factor, and kurtosis. Continuous wavelets transform (CWT) analysis is an effective tool for analysing bearing fault data, providing time, frequency, and amplitude details. CWT signatures obtained from the analysis are unique features that can be used to classify bearing faults. The signatures generated using CWT can be used as fault classification tools in ball bearing fault diagnosis^12^. Vibration data are utilised extensively in bearing fault detection as a result of the fact that it is reasonably simple to collect these data and that they contain a significant quantity of dynamic information about flaws. Because the operators make the decisions on the classification criteria, the fault detection accuracy that is based on statistical approaches is subject to a limitation^13^. highlights the potential application of machine learning (ML) methods for wear and fatigue fault detection and lifetime prognosis of sliding bearings using acoustic emission (AE) signals. The study showcases that abrasive and adhesive wear, as well as fatigue, are the main causes of bearing failure, and supervised learning ML algorithms can be used for diagnosis and prognosis in these cases^14^. proposes a medium Gaussian support vector machine (SVM) method for motor bearing fault diagnosis using machine learning, improving reliability and accuracy in fault estimation, detection, and identification. The proposed method constructs a feature space by extracting characteristics from vibration signals, and different Gaussian kernel functions (medium, fine, coarse) are analysed for their impact on SVM performance. Experimental data from the Case Western Reserve University Motor Bearing Data Centre^15^ validate the performance of the proposed method, including in noisy environments. The results demonstrate the effectiveness of the medium Gaussian SVM in classifying motor health and its potential for predictive maintenance analysis of electric vehicles. Convolution neural networks (CNNs) have gained significant traction in the field of machine health monitoring due to their extensive utilization^16–18^. The performance of the CNN classifier is highly influenced by the type of training data utilized in generating an image. In the context of bearing diagnosis, several types of training data forms were presented to enhance the performance of the CNN classifier. These forms include frequency data^19^, short-time Fourier transform spectrogram^20^, wavelet transform scalogram^21^, and vibration signal grey-scale image^22^. This study will focus on conducting a comparative analysis of the bearing fault classification using three machine learning techniques: support vector machine (SVM), Random Forest (RF), XGBoost, ensemble techniques and deep learning techniques: CNN (LeNet-5). In this study, we present a diagnostic approach aimed at improving the accuracy of categorization outcomes. Furthermore, we evaluate the effectiveness of this methodology and draw conclusions based on the findings. Feature Extraction: Since this work focuses on identifying various bearing faults using vibration signal involves sudden change in signal patterns in a very short time bust. This work focused on identifying feature vectors which perform well in these conditions. For a machine learning based classification approach, Mel-frequency cepstral coefficients (MFCCs) features are used to build the multiclass classifiers, and for a deep learning approach using CNN (LeNet-5), Wavelet Transform-based Scalograms are used to build the multiclass classifier model. Mel-frequency cepstral coefficients (MFCCs): Mel frequency cepstral coefficients (MFCCs) are widely recognized for their exceptional performance in detecting sudden short-term bursts in audio signals^23^. The efficacy of MFCCs in this regard can be attributed to their capacity to capture both spectral and temporal features of audio data. By initially segmenting the audio signal into short overlapping frames and subsequently applying the Mel filter bank, MFCCs excel at accentuating critical spectral components. This spectral representation, coupled with the discrete cosine transform (DCT) applied in the cepstral domain, serves to emphasise abrupt changes and transients in the audio signal. This work considers 13 coefficients, as they tend to capture the most important spectral information while keeping the dimensionality manageable for subsequent processing and classification tasks. Consequently, MFCCs are highly sensitive to short-duration bursts of sound energy, making them particularly adept at identifying and characterising such events. This attribute renders MFCCs invaluable in applications where the detection of sudden, transient audio phenomena is essential, such as in the domains of speech recognition, acoustic event detection, and anomaly detection in audio streams. Their robustness and discriminatory power in this context underscore their significance as a fundamental tool in audio signal analysis. The following diagram illustrates how MFCC features are extracted from the vibration signal. Add visual diagram Scalograms; Scalogram features, derived from the Continuous Wavelet Transform (CWT), exhibit promising potential for ball bearing fault detection in machinery. These features provide a comprehensive time-frequency representation of vibration signals, enabling the capture of both transient and frequency domain information crucial for identifying bearing faults. Scalograms excel in highlighting the spectral patterns and irregularities that often manifest as bearing defects, such as impact forces, harmonics, and non-stationary behaviour. Their time-frequency localization capability facilitates the detection of abrupt changes and transient events associated with faults, enhancing the sensitivity to fault signatures. Moreover, by offering a multi-resolution analysis, scalograms are adaptable to diverse bearing fault scenarios, from minor surface wear to catastrophic failures. However, the effectiveness of scalogram features in fault detection may depend on factors such as the choice of wavelet, scale parameters, and the nature of the vibration data. Therefore, their success hinges on careful configuration and consideration of the specific characteristics of the machinery under examination, making scalograms a valuable asset in the toolkit of condition monitoring and fault diagnosis in industrial systems. Mel-frequency cepstral coefficients (MFCCs); Random Forest: The Random Forest classifier is a prominent ensemble learning technique in machine learning, known for its robustness and high predictive accuracy. This algorithm, proposed by Leo Breiman in 2001, has found widespread application across various domains, including but not limited to bioinformatics, finance, and image recognition. It operates by constructing a multitude of decision trees during the training phase and subsequently aggregating their outputs to make predictions. This ensemble approach mitigates overfitting and enhances generalization by introducing randomness through bootstrapping and feature selection. The diversity of constituent trees ensures that the model captures intricate patterns in the data, making it particularly adept at handling complex and high-dimensional datasets. Moreover, the Random Forest classifier exhibits an innate capability to estimate feature importance, aiding in feature selection and interpretation of model results. As a result of these attributes, it has emerged as a versatile and valuable tool in the repertoire of machine learning practitioners^24^. Data Set: The CWRU Bearing Data Centre provides the input signals used in this analysis. Figure 2 depicts the experimental setup used to collect these measurements. A two horsepower (hp) Reliance Electric Motor fitted with a torque encoder and transducer makes up the experimental apparatus in question here. In the 12 o’clock position on the Drive End (DE) and Fan-End (FE) housing are three accelerometers. Meanwhile, both the DE and FE bearings are SKF deep-grove ball 6205−2RS JEM models. Electro-discharge machining was used to sow faults with sizes between 0.007 and 0.028 inches. A 16-channel data recorder sampled at 12 kHz and 48 kHz was used to record vibration signals under four different loads and rotational speeds (from 1797 to 1700 rpm), which were post-processed in a MATLAB environment. All data files are in MATLAB (*.mat) format. DE, FE, and BA data are all recorded and can be found in separate files. Data was gathered at 12k and 48k samples per second during the drive-end bearing studies. The data from the fans was sampled at a rate of 12k samples per second. A rate of 48k samples per second was used for the normal baseline. There are a total of 161 records in the dataset, and they may be broken down into four distinct categories: 48k normal-baseline, 48k drive-end fault, 12k drive-end fault, and 12k fan-end fault. Ball bearing (B) fault data, inner-race fault data, and outer-race fault data are all included in the respective groups. The outside race faults are further categorized as ’centred’ (fault at the 6.00 o’clock position), ’orthogonal’ (fault at the 3.00 o’clock position), and ’opposite’ (fault at the 12.00 o’clock position) depending on their placement in relation to the load zone.

The work in^25^ proposes an approach to identify ball bearing fault leveraging Wavelet Packet Transform (WPT) for signal processing and k-Nearest Neighbour (KNN) to classify type of fault. The CWRU vibration dataset were used for feature extracted from statistical technique and Hjorth parameters. An accuracy of 99.65% was achieved with 4096 samples with the combination of four sets of feature vector combined with RMS, kurtosis, spectral entropy, and Hjorth parameters. The combination of WPT together with signal decomposition yields effective isolation of frequency related to faults, improving the efficacy of classification.

The work in^26^ presents a deep learning based approach to identify the ball bearing faults. The proposed approach combines the traditional classifier SVM, stacked sparse autoencoder (SSAE) networks with local cyclic mapping (LCM), and a SoftMax classifier to enhance the accuracy of fault detection. Both normal and faulty samples of vibrating data are taken for train and test the model from the bearing test bench operating at 1797 RPM. The proposed approach of combining SSAE with LCM improved the result by 0.6% compared with traditional SSAE technique. The work also emphasizes on model overfitting due to increase in the hidden layers.

The work in^27^ focuses on addressing the challenges in identification of ball bearing faults for imbalanced dataset where normal samples largely out sample the faulty samples. To address the issue at hand authors proposed Deep Transfer Learning (DTL) model combined with a Res2Net-Convolutional Block Attention Mechanism (CBAM). The CBAM improves the feature extraction by focusing on critical faulty features, thereby reducing noises. The DTL reduces negative migration effects by transferring pretrained weights from balanced to imbalanced datasets. the CWRU and Paderborn University bearing datasets were used to conduct the experiments achieving accuracies of 95.8% and 95.85% respectively, highlighting the efficacy of the proposed technique under severe imbalance and variable load conditions.

The work in^28^ developed a hybrid CNN-LSTM architecture for diagnosing progressive wear in rolling element bearings via vibration signals. Vibration recordings were taken at 0, 1000, and 2000 h, showing increased amplitudes at inner race defect. Raw signals are contaminated by non-stationary noise, were processed through Empirical Mode Decomposition (EMD) to generate 12 Intrinsic Mode Functions (IMFs). The optimal IMF was identified using the maximum energy ratio approach, effectively reducing noise while retaining fault-related impulses. This enhanced data served as input to a multi-scale CNN with parallel branches and dense softmax output. The model achieved 99.65% training accuracy, 98.27% test accuracy, and F1-score of 0.99 outperforming standalone CNN, LSTM, and prior studies, the framework offers reliable early fault detection for industrial predictive maintenance.

The work in^29^ conducted a comparative analysis of neural network models—artificial neural network (ANN), 1-D convolutional neural network (CNN), multi-input 1-D CNN, and 2-D CNN—for diagnosing faults in rolling element bearings with naturally occurring operational defects. The study utilized a test rig where NJ307ECP bearings operated under a constant 800 rpm speed and 1.5 kN radial load for 2000 h, inducing progressive surface wear like pitting and spalling. Vibration signals were acquired at intervals using a DEWESoft system at 20 kHz sampling rate, revealing escalating fault signatures primarily on the inner race. Raw data underwent preprocessing with windowing and stride adjustments to form subsequences, split into 70/15/15 for training, testing, and validation. Models were built in Keras/TensorFlow, employing Adam optimizer and categorical cross-entropy loss. The multi-input 1-D CNN, featuring parallel convolutional branches for complementary feature extraction, achieved the highest accuracy at 97%, with F1-score of 0.97, outperforming ANN (89%), standard 1-D CNN (93%), and 2-D CNN (95%). Confusion matrices and t-SNE visualizations confirmed robust fault categorization into healthy, early, and severe stages. This work highlights the superiority of multi-branch architectures over single-path models for handling variable operating conditions, surpassing prior efforts in natural defect scenarios, advancing predictive maintenance in rotating machinery.

The study contributes to SDG 9 by presenting a novel acoustic-driven machine learning framework aimed at improving the reliability and durability of industrial operations. In this study, the term ‘acoustic vibration signals’ refers specifically to vibration data acquired through accelerometers, which represent mechanical vibration rather than airborne acoustic signals^30^. Through accurate identification of bearing defects at an early stage, the approach minimizes unplanned failures and reduces costly disruptions across manufacturing and transportation sectors. By leveraging deep learning for predictive maintenance, this work promotes sustainable industrial development while driving forward technological innovation. The simultaneous stacking of fan-end and drive-end scalograms was adopted to capture cross-sensor complementary information, as both sensor locations record different aspects of the same fault event. Stacking creates a multi-channel representation that allows the CNN to learn correlated temporal–frequency patterns that may not be visible in single-sensor inputs^31^.

Following are the contribution of the proposed work:

- The study introduces a novel framework for ball bearing fault detection using acoustic vibration signals. Unlike traditional vibration-only methods, the proposed method leverages sound-based data acquisition, improving the fault detection which are sensitivity to subtle mechanical anomalies.
- Two distinct analytical pipelines were implemented:
  - Machine Learning (ML): Using MFCC features with Random Forest and XGBoost classifiers.
  - Deep Learning (DL): Using scalogram images obtained from the Continuous Wavelet Transform (CWT) which are processed through LeNet-5 CNN model.

This dual approach allows for a thorough comparison of numerical feature-based with image-based learning models.

- An wavelet-based time–frequency representation (scalogram) combined with LeNet-5 CNN, enabling localized identification of transient and non-periodic fault patterns.

## Methodology

**Fig. 1:**
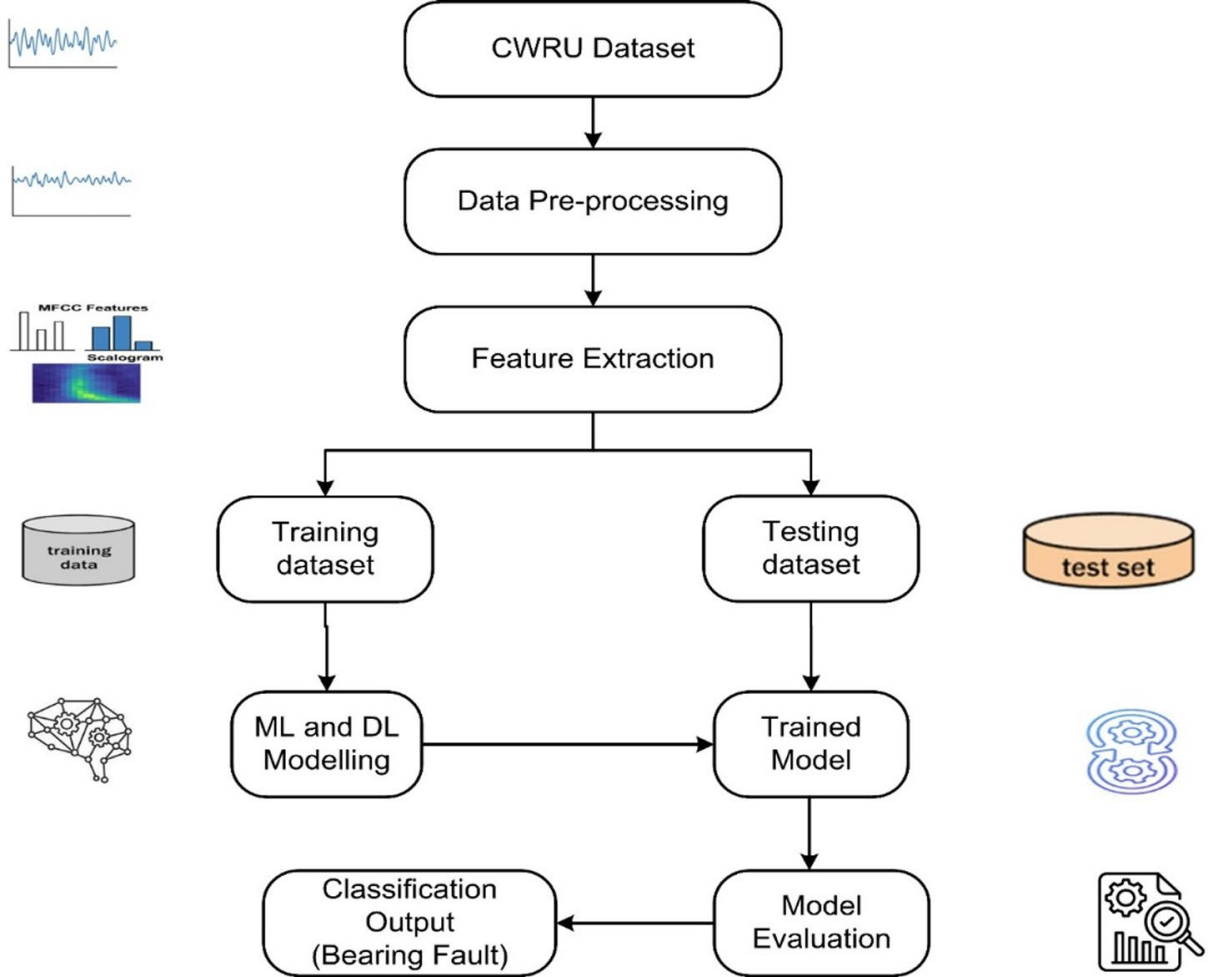
Proposed workflow for ball bearing fault detection system using CWRU dataset, with data-preprocessing, feature extraction for ML/DL technique, ML/DL modelling and model evaluation metrics.

This section outlines the methodology used to develop the proposed ML and DL based framework with CWRU dataset, data preparation, feature extraction, system workflow for class prediction and evaluation metrics applied to measure the model performance illustrated in the Fig. 1.

### Dataset

The Case Western Reserve University (CWRU) Bearing Dataset is a widely used benchmark dataset for the diagnosis of faults in rolling element bearings that occur during machine health monitoring. A 2 horsepower Reliance Electric motor was utilized to conduct experiments. Single point faults were created through Electro-Discharge Machining (EDM) in the inner race (IR), balls (B), and outer race (OR) of ball bearings. The faults were placed at either the drive end (DE) or fan end (FE) of the motor. Acoustic vibration signals were collected from accelerometers located at the drive end (DE), fan end (FE), and motor base. Sampling occurred at 12 kHz for most cases (48 kHz for some DE cases), while the motor load varied from 0 to 3 horsepower which corresponds to a range of rotational speeds of about 1720–1797 RPM. For DE faults, the severity of faults ranged from 0.007 to 0.028 inches, while FE faults were allowed to reach a maximum severity of 0.04 inches. In addition to the varying degrees of faults, the dataset contains normal baseline data. Due to the manner in which vibration signals propagate through a medium, the recorded signals have been referred to as acoustic vibration even though they indicate mechanical vibration.

### Mel frequency cepstrum

The Mel scale (or melody scale) is a measurement scale of pitches judged by listeners positioned at equal distances from one another. The association between the Mel scale and normal frequency measurement is defined by assigning a perceptual pitch of 1000 mels to a 1000 Hz frequency. Mel frequency cepstrum (MFC) is a cosine transform-based representation of the power spectrum of a sound with frequencies measured using the Mel scale. The Mel cepstrum yields a compact representation of a sound wave. MFC is collectively represented by Mel Frequency Cepstrum Coefficients computed for a signal as below.

- Compute the Fourier transform of the signal.
- Map the powers of the Fourier spectrum onto Mel scale using a suitable selection of overlapping windows.
- Take the logarithm of the powers at each Mel frequency.
- Take the discrete cosine transform of the list of Mel log powers.
- The amplitudes of the above spectrum form the MFCC coefficients Mel spectrogram is a color image-based representation of MFCC coefficients where each color represents relative power intensity of the corresponding Mel frequency.

A sample Mel spectrum of a signal used for ball bearing fault detection is shown in Fig. 1.

**Fig. 2:**
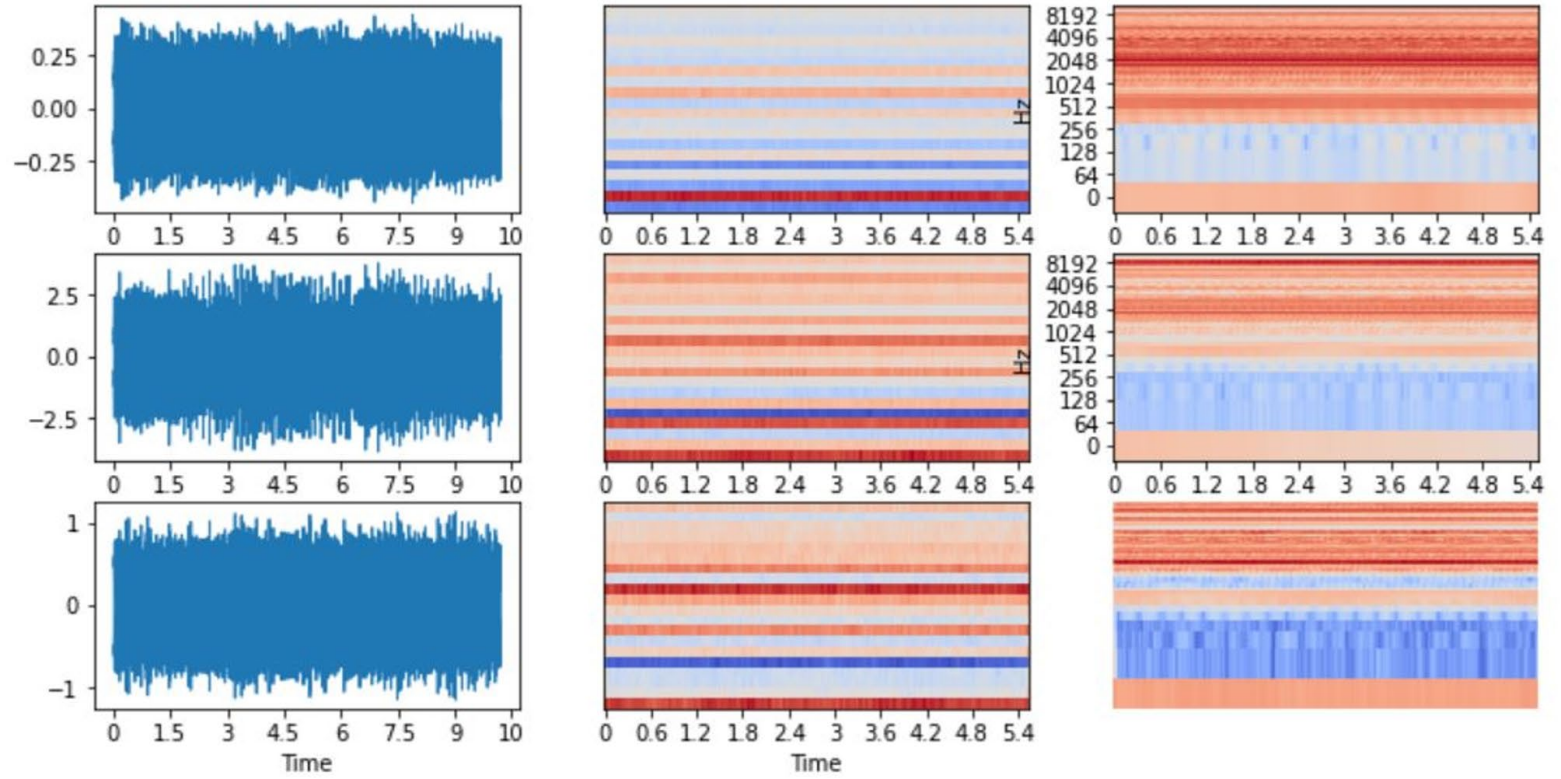
Comparison of time-domain waveforms and corresponding Mel spectrograms for three audio samples. Left column: raw audio signals exhibiting varying degrees of noise and amplitude fluctuations over a 10-second duration.

For training the fault classification model, the signal corresponding to each fault class is considered, and the first twenty Mel frequency cepstrum coefficients are computed. Later, the collection of these coefficients was fed to the random forest and XGBoost classifiers to learn a classification model which can be used to categorize any upcoming signal into one of the classes. However, the Mel spectrogram, being a Fourier transform-based representation, can only capture repeatedly occurring frequencies. So, the spectrum may not be very effective in detecting sudden short-term bursts in the signal, which may be unique to the vibration of each fault class of bearings. With this assumption, it is proposed to apply the short-term based continuous wavelet transforms to overcome the shortcomings of the Mel spectrogram.

### Continuous wavelet transform of acoustic vibration signals

Wavelets are small waves or brief oscillations which are popularly used to detect important features in a signal. The detection is made possible by convolving the wavelet with the original signal and analysing the resulting waveform. A wavelet correlates with a signal if a portion of the signal is similar and accentuates such similar portions. Various basic mother wavelet forms exist in the signal processing literature. Each wavelet can be scaled along the amplitude axis or translated along the time axis to facilitate approximate matching between a signal and the wavelet. Here, in the present study, a specific mother wavelet, called the Morlet (or Dabor) is used and is shown in Fig. 2.

**Fig. 3:**
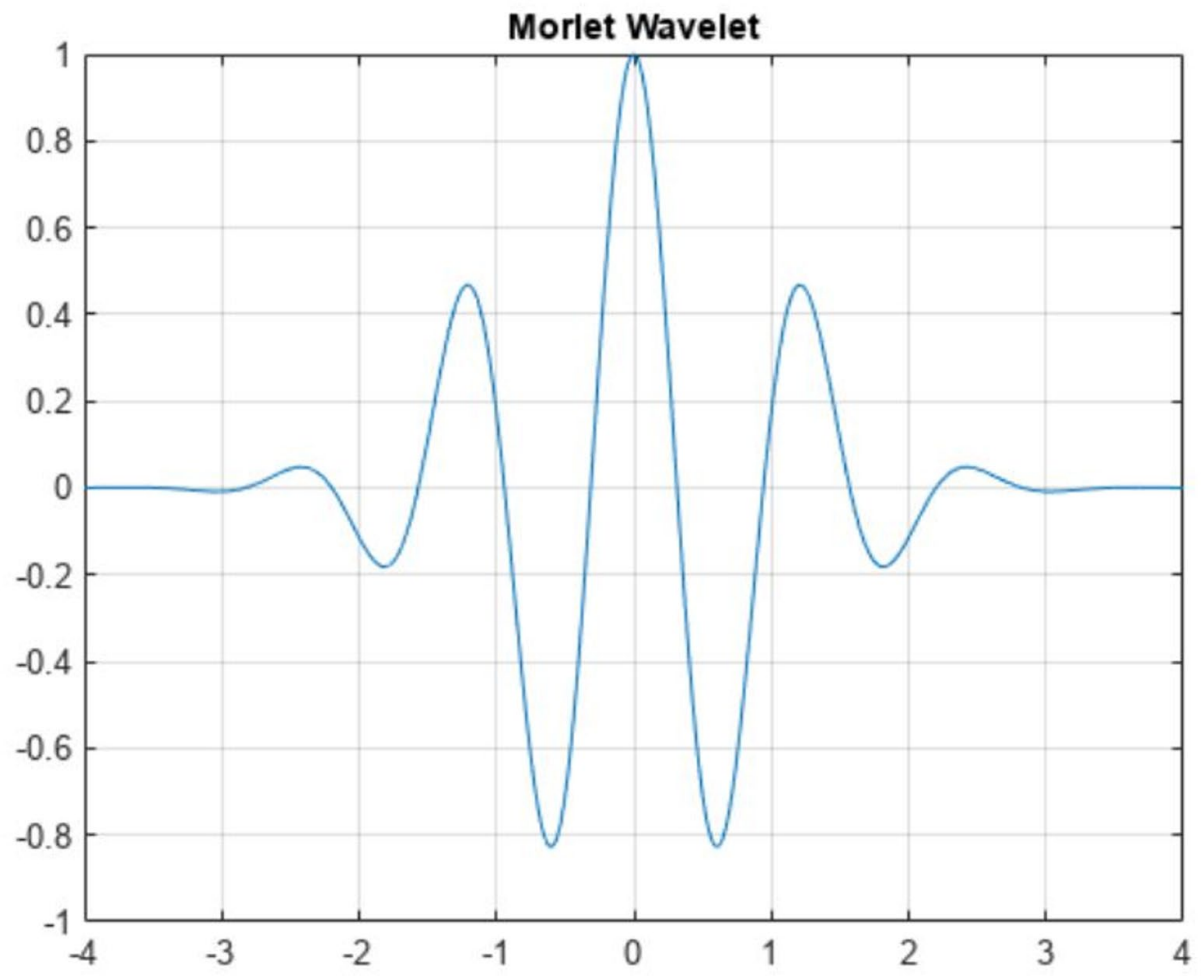
Time-domain representation of a Morlet wavelet.

A Continuous Wavelet Transform (cwt) of a signal for a given mother wavelet is a representation of the signal in terms of the mother wavelet, the coefficients of which give the magnitude of correlation between the mother wavelet and the portion of the signal, under different possible scaling or translational operations. These coefficients can be represented in a colour image called a scalogram. A list of sample scalograms for different fault classes is given in Fig. 3.

**Fig. 4:**
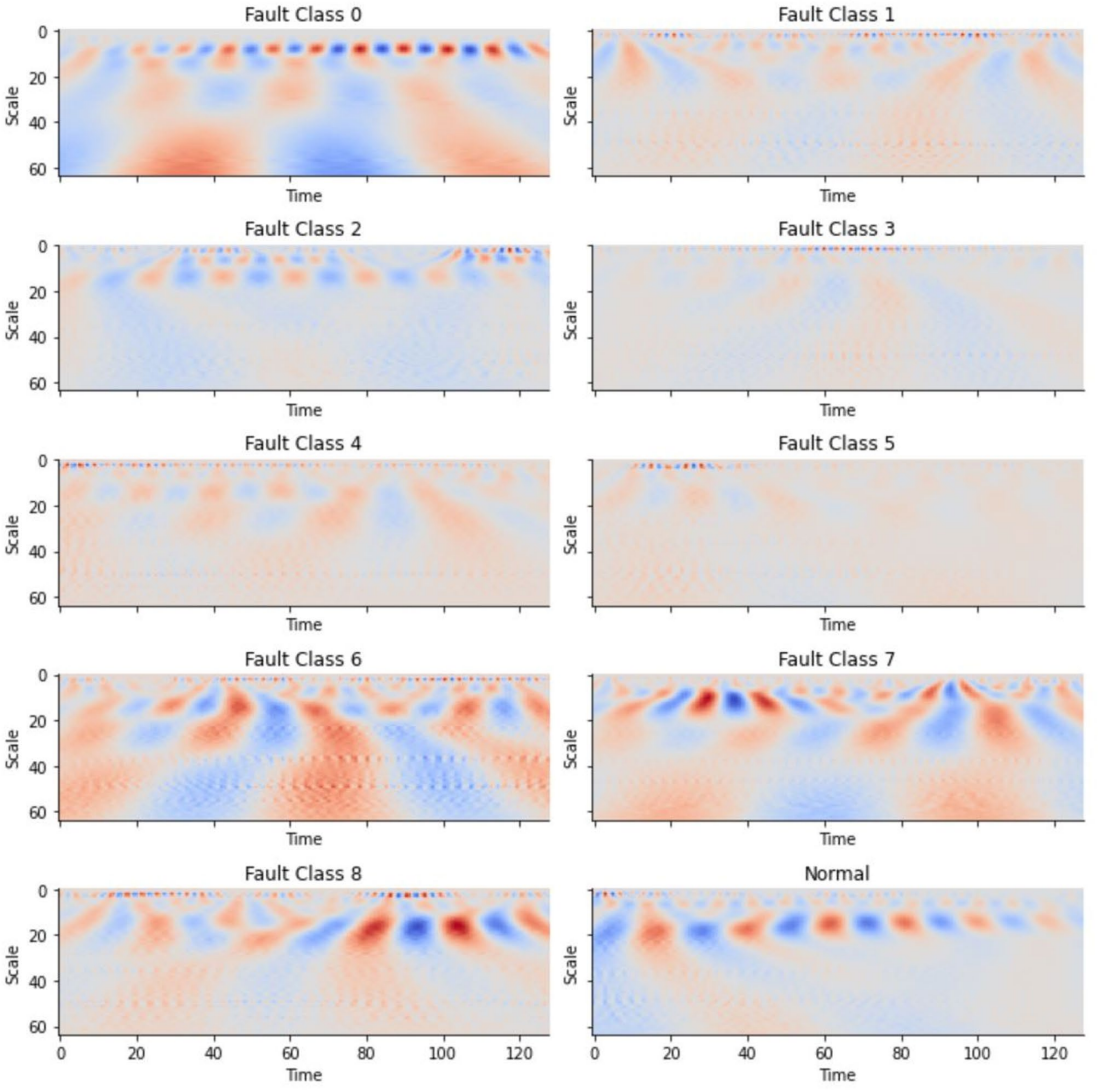
Continuous Wavelet Transform (CWT)-based scalogram representations of acoustic vibration signals for various bearing fault classes (Class 0–8) and the normal condition.

A signal is split into equally sized windows, and built scalograms for the portion of the signal in each window. Because of the collection of multiple vibration signals from multiple locations, a window-size scalogram for each signal is constructed. Since multiple signal measurements confirm with confidence the presence or absence of a fault, all the multiple signals together in each window are considered and generate scalograms for each signal within the window and use that stacked collection in the deep learning network, LeNet-5.

### LeNet-5 architecture

This section briefly describes the architecture of LeNet-5, a convolutional neural network developed for image classification. LeNet is a convolutional neural network made up of 7 layers^32,33^. The layers include three convolutional layers, two subsampling layers, and two fully connected layers. After the second convolution and subsampling, the output is flattened to a single vector, creating a long layer of hidden nodes, which in turn is fully connected to a following sequence of fully connected layers with a lower number of nodes. The LeNet architecture is presented in Fig. 4 and hyper parameters used for the architecture is presented in the Table 1. Hyperparameters were tuned through guided manual experimentation over a limited set of values informed by prior studies, rather than through an exhaustive grid or Bayesian search^34^.

**Fig. 5:**
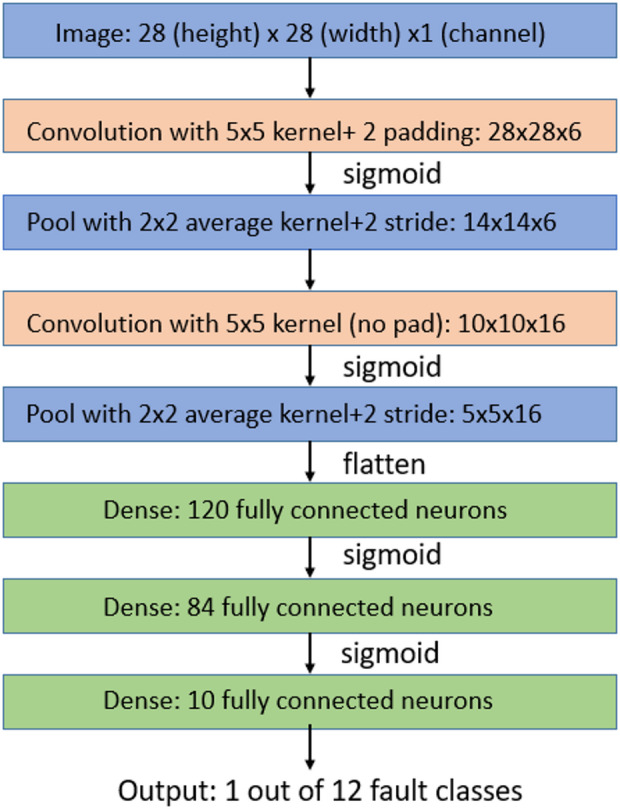
Architecture of the proposed convolutional neural network (CNN) for fault classification.

**Table 1:** Parameters of Lenet-5 CNN Architecture.

Layer Type	Description	Kernel	Stride	Padding	Output Dimension	Activation Function
Input Layer	Input Image (Scalogram)	–	–	–	28 × 28 × 1	–
Convolution Layer 1	Convolution with 5 × 5 kernel	6 filters	1	2	28 × 28 × 6	Sigmoid
Pooling Layer 1	Average Pooling	2 × 2	2	–	14 × 14 × 6	–
Convolution Layer 2	Convolution with 5 × 5 kernel	16 filters	1	None	10 × 10 × 16	Sigmoid
Pooling Layer 2	Average Pooling	2 × 2	2	–	5 × 5 × 16	–
Flatten Layer	Converts 3D tensor to vector	–	–	–	400	–
Fully Connected Layer 1	Dense Layer	120 neurons	–	–	120	Sigmoid
Fully Connected Layer 2	Dense Layer	84 neurons	–	–	84	Sigmoid
Output Layer	Dense Layer (Classification)	12 neurons (mapped to 12 fault classes*)	–	–	1 × 12	Softmax/Sigmoid

## Results & discussion

In this section, classification results of the two models described above are reported. The summary of the evaluation protocol is reported in the Table 2.

**Table 2:** Summary of evaluation protocol.

Step	Description
Dataset	Case Western Reserve University (CWRU) Bearing Dataset
Signal Segmentation	Non-overlapping 128 ms window segments
Data Split	70% training, 30% testing with stratified by fault type
Validation	Repeated random sub-sampling with 5 iterations
Leakage Control	Non-overlapping windows; independent test segments^35^
Metric Reported	Accuracy, Precision, Recall, and F1-score

The hardware and the software configuration used in the proposed work is reported in Table 3.

**Table 3:** Hardware and software configuration used for proposed work.

Component	Specification/Version
Operating System	Ubuntu 22.04 LTS
Platform	Jupyter Hub
GPU	NVIDIA A100 80GB PCIe Gen
CPU	intel Xeon Gold 6330 28 C 205 W 2.0 GHz Processor
RAM	32 × 16 = 512gb
Python Version	3.10.12
Deep Learning Framework	PyTorch
Audio Library	Torch Audio
Dataset Handling	Numpy, Pandas

**Fig. 6:**
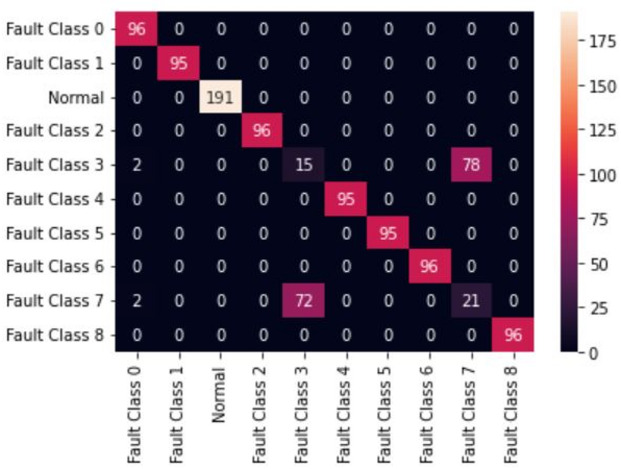
Confusion matrix for random forest model (Accuracy 85.3%).

**Fig. 7:**
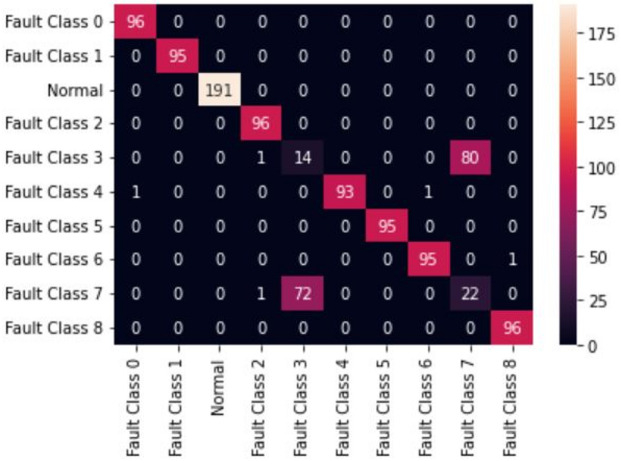
Confusion matrix for XGBoost model (Accuracy: 85.00%).

**Fig. 8:**
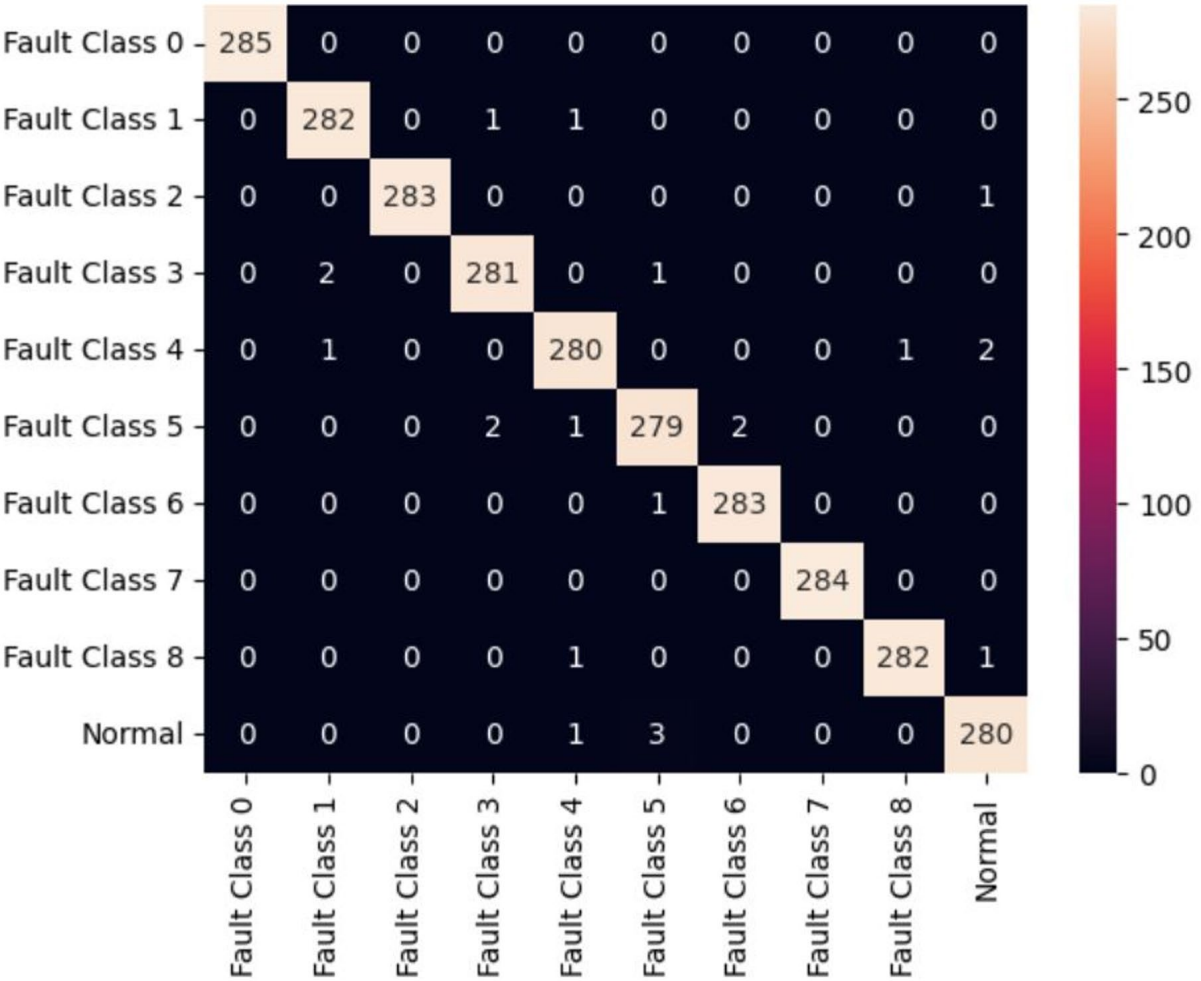
Confusion Matrix for LeNet-5 Model (Accuracy: 99.23%).

The first model uses 20 MFCC coefficients derived for both Fan-end and Drive-end signals as feature inputs to two individual classification models. The difficulty of MFCCs in capturing transient bursts was inferred from the observed smoothing of MFCC patterns across classes, in contrast to the localized high-energy structures visible in the corresponding scalograms^36^. The classification models considered are Random Forest and XGBoost. These models resulted in a classification accuracy of 85%. Both models offer similar performance, as can be seen in Figs. 5 and 6. The confusion matrix of these models can be seen in Figs. 5 and 6. In the second model, instead of the original numerical signals, the scalogram images of individual signals obtained from the Morlet wavelet are used in the classification model. The full length (121,256 ms) Fan-end and Drive-end signals are split into smaller signals of window size 128 ms, and build scalograms for each window-size subsignal. A 128 ms non-overlapping window was selected based on preliminary tests that ensured sufficient time–frequency resolution for generating stable scalograms^37^. These scalograms are stacked together to create a 3D input array and feed those stacked images into the LeNet-5 neural network. The network is trained on randomly picked scalograms. 70 per cent of the total number of scalograms constitutes the training set, and the remaining constitutes the test set. The LeNet-5 CNN was optimized for the CWT-based scalogram inputs to achieve high accuracy with efficient computation. The model was trained using the Adam optimizer (learning rate = 0.001), a batch size of 32, and 50 epochs. A dropout rate of 0.3 was applied to prevent overfitting, with categorical cross-entropy as the loss function and Softmax for multi-class output. Early stopping was used to halt training upon convergence. LeNet-5 could result in an additional 14% accuracy, giving the final accuracy of 99.23%. To ensure that the reported accuracy was not a result of overfitting, the model was trained using stratified data splits with repeated random sub-sampling validation, early stopping, and dropout regularization. The training and validation losses were monitored throughout training and showed consistent convergence without divergence or instability. These checks indicated that the model generalized well to unseen test segments^38^. The wavelet-based image models seem to have an advantage over the numerical signal-based classification models. The confusion matrix for the LeNet-5-based model is given in Fig. 7. The Random Forest (RF) model was optimized to balance complexity and performance. Key parameters - n_estimators (100–500) and max_depth (10–30) - were tuned, and both Gini and entropy criteria were tested. The best configuration, with 200 estimators, max_depth of 20, and Gini criterion, achieved the highest accuracy while minimizing overfitting and training time, ensuring stable classification across all bearing fault categories. The XGBoost model was fine-tuned to improve generalization and reduce overfitting. Key parameters - learning rate (0.01–0.2), max_depth (6–10), and n_estimators (100–500)- were optimized through experimentation, with a subsample ratio of 0.8 applied for robustness. The best configuration, learning rate of 0.1, max_depth of 8, and n_estimators of 300, achieved a balanced bias–variance trade-off and produced a smooth, stable learning curve with minimal error. Table 4 reports the accuracy comparison over the original implementations of Random Forest and XG Boost presented in^39^. The present work considered only the classes for which full vibration data is available. Table 5 gives the accuracies, and Fig. 8 gives the performance of the various DL and signal processing methods and pure ML methods. While all the methods offer comparable performance, the signal processing and image-based Deep Learning method presented in this study has an edge over others. Further, visual methods have the additional advantage that it is easy to detect any anomalous pattern visually in real time.

**Table 4:** Performance comparison with the State-of-the-art ML Methods.

Method	Accuracy	Precision	Recall	F1 Score
CWTI - DL	99.23	99.21	99.28	99.40
Random Forest	98.17	98.30	98.20	98.30
XG Boost	98.08	98.10	98.00	98.10

**Table 5:** Performance comparison with various deep learning (DL), signal processing (SP), and machine learning (ML) Methods.

Method	Type	Accuracy	% Training data
CWTI – DL	SP + DL	99.23	70
Compx – WL – DL	SP + DL	94.38	67
WL DL (num)	DL	95.20	70
Ensemble DL	DL	99.15	67
RF	ML	98.17	85
XG Boost	ML	98.08	85
SVM	ML	97.51	85
Ensemble	ML	97.90	80

**Fig. 9:**
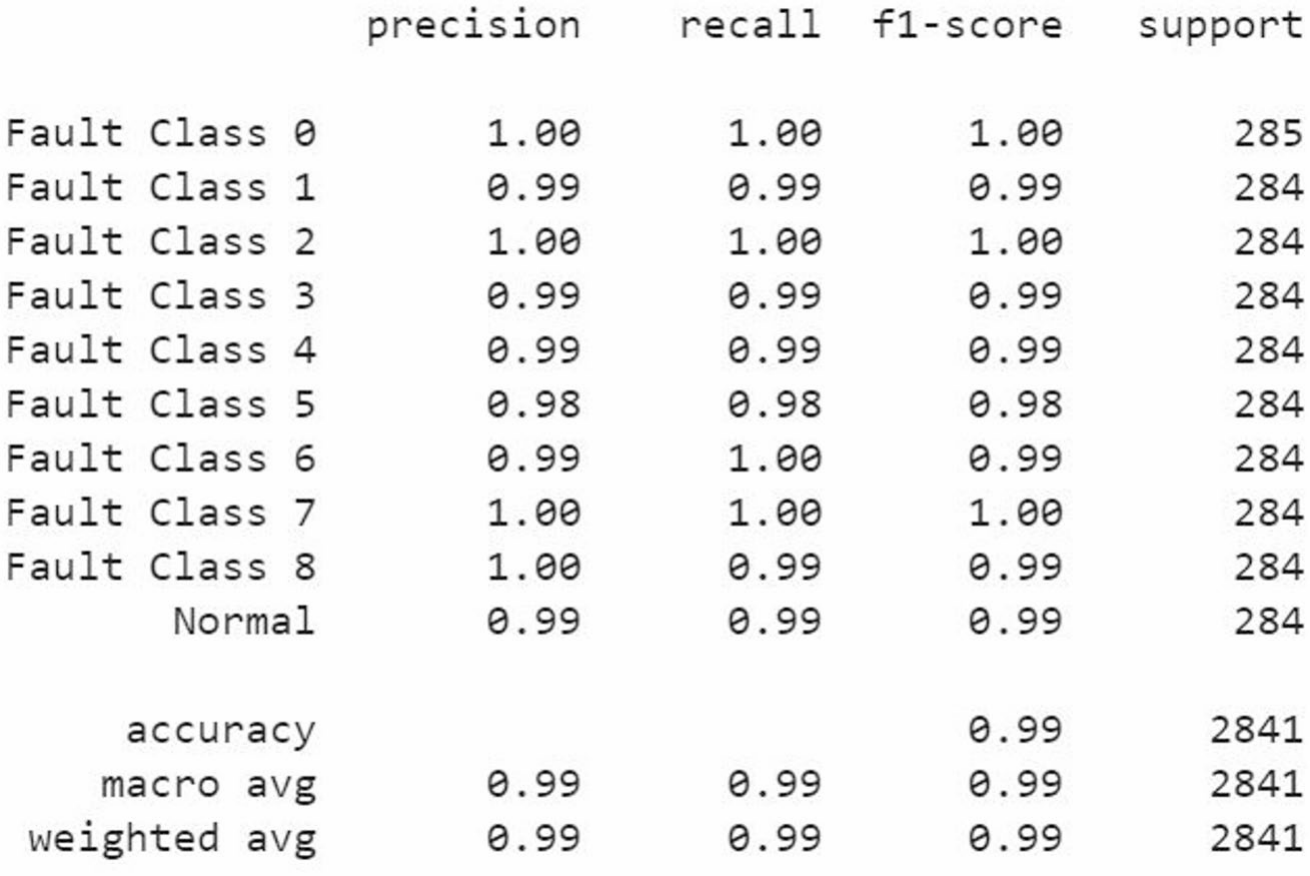
Performance of the proposed LeNet-5 Model (Accuracy: 99.23%).

Figure 9 demonstrates that the proposed LeNet-5 model achieves consistently high classification performance across all fault classes and the normal condition, with precision, recall, and F1-scores predominantly around 0.99–1.00. The overall accuracy of 99.23% indicates the strong discriminative capability of the model in correctly identifying multiple fault conditions with minimal misclassification. Furthermore, the close agreement between macro-average and weighted-average metrics confirms the robustness and balanced performance of the model across all classes.

### Conclusion and future scope

In this work, a continuous wavelet transform-based convolutional neural network classifier for fault identification in bearings is presented. The diverse set of ML and DL techniques explored in this work provides insight into the selection of the most appropriate model for specific maintenance scenarios. The proposed model has a higher prediction accuracy (= 99.23%) than the numerical signal-based classification models. The proposed work uses only CWRU dataset may not fully represent the real-world industrial environment. As an extension of this work, future research will involve experimentation with more complex and deeper networks capable of handling a broader range of fault detection tasks together with additional datasets which covers broader real work industrial environment.

The stacked scalogram representation was chosen to allow the network to learn cross-sensor temporal–frequency correlations; however, a quantitative comparison with single-sensor or late-fusion approaches remains a promising direction for future work.

## Data Availability

The datasets used and/or analysed during the current study available from the corresponding author on reasonable request.

## References

[1] Boumahdi, M., Rechak, S. & Hanini, S. Analysis and prediction of defect size and remaining useful life of thrust ball bearings: modelling and experiment procedures. *Arab. J. Sci. Eng.*10.1007/s13369-017-2550-y (2017).

[2] Tyagi, S. & Panigrahi, S. K. A DWT and SVM based method for rolling element bearing fault diagnosis and its comparison with artificial neural networks. *J. Appl. Comput. Mech.*10.22055/jacm.2017.21576.1108 (2017).

[3] Choudhary, A., Goyal, D., Shimi, S. L. & Akula, A. Condition monitoring and fault diagnosis of induction motors: A review. *Arch. Comput. Methods Eng.*10.1007/s11831-018-9286-z (2019).

[4] Gangsar, P. & Tiwari, R. Signal based condition monitoring techniques for fault detection and diagnosis of induction motors: A state-of-the-art review. *Mech. Syst. Signal Process.*10.1016/j.ymssp.2020.106908 (2020).

[5] Altaf, M. et al. Automatic and Efficient Fault Detection in Rotating Machinery using Sound Signals. *Acoust. Aust* **47**(2), 125–139. 10.1007/s40857-019-00153-6.

[6] Dave, V., Singh, S. & Vakharia, V. Diagnosis of bearing faults using multi fusion signal processing techniques and mutual information. *Indian J. Eng. Mater. Sci.* **27**(04), 10.56042/ijems.v27i4.44862 (2020).

[7] D, J. R. M. S. N. S. and M. B, Robust And Efficient Classification of Fault in Ball Bearings Using Machine Learning, in *8th International Conference on Smart Structures and Systems (ICSSS)*, Apr. 2022, pp. 1–7, Apr. 2022, pp. 1–7, (2022). 10.1109/ICSSS54381.2022.9782206

[8] Delgado, M., Cirrincione, G., Garcia, A., Ortega, J. A. & Henao, H. Accurate bearing faults classification based on statistical-time features, curvilinear component analysis and neural networks, in *IECON –38th Annual Conference on IEEE Industrial Electronics Society*, Oct. 2012, pp. 3854–3861, Oct. 2012, pp. 3854–3861, (2012). 10.1109/IECON.2012.6389596

[9] Sharma, K., Goyal, D. & Kanda, R. Intelligent Fault Diagnosis of Bearings based on Convolutional Neural Network using Infrared Thermography, *Proc. Inst. Mech. Eng. Part J J. Eng. Tribol.*, vol. 236, no. 12, pp. 2439–2446, Dec. (2022). 10.1177/13506501221082746

[10] Aasi, A., Tabatabaei, R., Aasi, E. & Jafari, S. M. Experimental investigation on time-domain features in the diagnosis of rolling element bearings by acoustic emission. *JVC/Journal Vib. Control*. 10.1177/10775463211016130 (2022).

[11] Prakash Kumar, J., Chauhan, P. S. & Prakash Pandit, P. Time domain vibration analysis techniques for condition monitoring of rolling element bearing: A review, *Mater. Today Proc.*, (2022). 10.1016/j.matpr.2022.02.550

[12] Saxena, M., Bannet, O. O., Gupta, M. & Rajoria, R. P. Bearing fault monitoring using CWT based vibration signature. *Procedia Eng.* **144**, 234–241. 10.1016/j.proeng.2016.05.029 (2016).

[13] König, F., Jacobs, G., Stratmann, A. & Cornel, D. Fault detection for sliding bearings using acoustic emission signals and machine learning methods. *IOP Conf. Ser. Mater. Sci. Eng.* **1097**(1), 012013. 10.1088/1757-899X/1097/1/012013.

[14] Lin, S. L. Application of Machine Learning to a Medium Gaussian Support Vector Machine in the Diagnosis of Motor Bearing Faults. *Electronics* **10**, 2266. 10.3390/electronics10182266.

[15] Case Western Reserve University Bearing Data Center (Year) Bearing data set. Case School of Engineering. Available at: https://engineering.case.edu/bearingdatacenter/download-data-file (Accessed: 25 January 2023).

[16] Pinedo-Sánchez, L. A., Mercado-Ravell, D. A. & Carballo-Monsivais, C. A. Vibration analysis in bearings for failure prevention using CNN. *J. Brazilian Soc. Mech. Sci. Eng.*10.1007/s40430-020-02711-w (2020).

[17] Shen, S. et al. A physics-informed deep learning approach for bearing fault detection. *Eng. Appl. Artif. Intell.* **103**, 104295. 10.1016/j.engappai.2021.104295.

[18] Zhang, Y. et al. A new method for diagnosing motor bearing faults based on Gramian angular field image coding and improved CNN-ELM. *IEEE Access.*10.1109/ACCESS.2023.3241367 (2023).37608804

[19] Verstraete, D., Ferrada, A., Droguett, E. L., Meruane, V. & Modarres, M. Deep learning enabled fault diagnosis using time-frequency image analysis of rolling element bearings. *Shock Vib.*10.1155/2017/5067651 (2017).

[20] Tayyab, S. M., Chatterton, S. & Pennacchi, P. Image-Processing-Based intelligent defect diagnosis of rolling element bearings using spectrogram images. *Machines*10.3390/machines10100908 (2022). 10.3390/s22052026 PMC8914858 35271173

[21] Neupane, D., Kim, Y. & Seok, J. Bearing fault detection using scalogram and switchable Normalization-Based CNN (SN-CNN). *IEEE Access.*10.1109/ACCESS.2021.3089698 (2021).

[22] Mishra, R. K., Choudhary, A., Fatima, S., Mohanty, A. R. & Panigrahi, B. K. A Fault Diagnosis Approach Based on 2D-Vibration Imaging for Bearing Faults. *J. Vib. Eng. Technol.*10.1007/s42417-022-00735-1.

[23] Abdul, Z. K. & Al-Talabani, A. K. Mel frequency cepstral coefficient and its applications: A review. *IEEE Access.*10.1109/ACCESS.2022.3223444 (2022).

[24] Parmar, A., Katariya, R. & Patel, V. A Review on Random Forest: An Ensemble Classifier, in Lecture Notes on Data Engineering and Communications Technologies, pp. 758–763. (2019).

[25] Mufazzal, S., Muzakkir, S. M. & Khanam, S. Intelligent evaluation of ball bearing health degradation using wavelet packet transform and k-Nearest neighbor, in Lecture Notes in Mechanical Engineering, pp. 367–378. (2023). 10.1007/978-981-19-2188-9_34

[26] Zhang, B. Rolling Bearing Fault Detection System and Experiment Based on Deep Learning. *Comput. Intell. Neurosci.* 1–10. 10.1155/2022/8913859 (2022). 10.1155/2022/8913859 PMC9532076 36203721

[27] Wang, H. & Zhang, X. Fault Diagnosis Using Imbalanced Data of Rolling Bearings Based on a Deep Migration Model,*IEEE Access.*, **12**, 5517–5533, doi: 10.1109/ACCESS.2024.3350785. (2024).

[28] Sahu, D., Dewangan, R. K., Matharu, S. P. S. & Hybrid CNN-LSTM model for fault diagnosis of rolling element bearings with operational defects. *Int. J. Interact. Des. Manuf.* **19**, 5737–5748. 10.1007/s12008-024-02165-7 (2025).

[29] Sahu, D., Dewangan, R. K. & Matharu, S. P. S. Fault diagnosis of rolling element bearing with operationally developed defects using various convolutional neural networks. *J. Fail. Anal. Preven*. **24**, 1310–1132. 10.1007/s11668-024-01919-5 (2024).

[30] Salunkhe, V. G., Desavale, R. G. & Jagadeesha, T. Experimental Frequency-Domain vibration based fault diagnosis of roller element bearings using support vector Machine. ASME. *ASME J. Risk Uncertain. Part. B June*. **7** (2), 021001. 10.1115/1.4048770 (2021).

[31] Salunkhe, V. G., Khot, S. M., Yelve, N. P., Jagadeesha, T. & Desavale, R. G. January 13, Rolling Element Bearing Fault Diagnosis by the Implementation of Elman Neural Networks With Long Short-Term Memory Strategy. ASME. J. Tribol. August 2025; 147(8): 084301. (2025). 10.1115/1.4067382

[32] Salunkhe, V. G., Desavale, R. G., Khot, S. M., Yelve, N. P. & February 26, Identification of Bearing Clearance in Sugar Centrifuge Using Dimension Theory and Support Vector Machine on Vibration Measurement. ASME. ASME J Nondestructive Evaluation. May 2024; 7(2): 021003. (2024). 10.1115/1.4064613

[33] Salunkhe, V. G. & Desavale, R. G. February 23, An Intelligent Prediction for Detecting Bearing Vibration Characteristics Using a Machine Learning Model. ASME. ASME J Nondestructive Evaluation. August 2021; 4(3): 031004. (2021). 10.1115/1.4049938

[34] Salunkhe, V. G., Khot, S. M., Desavale, R. G. & Yelve, N. P. July 26, Unbalance Bearing Fault Identification Using Highly Accurate Hilbert–Huang Transform Approach. ASME. ASME J Nondestructive Evaluation. August 2023; 6(3): 031005. (2023). 10.1115/1.4062929

[35] Salunkhe, V. G., Khot, S. M., Yelve, N. P., Desavale, R. G. & Raut, A. S. May 20, Vibration Dynamic Analysis of the Bearing Parameters in Steam Turbine Bearing Systems in Sugar Refinery. ASME. J. Tribol. January 2026; 148(1): 014301. (2025). 10.1115/1.4068559

[36] Salunkhe, V. G., Desavale, R. G., Khot, S. M. & Yelve, N. P. April 3, 2023). A novel incipient fault detection technique for roller bearing using deep independent component analysis and variational modal Decomposition. ASME. *J. Tribol July*. **145** (7), 074301. 10.1115/1.4056899 (2023).

[37] Verdhan, V. Image Classification Using LeNet. In: Computer Vision Using Deep Learning Apress, Berkeley, CA.2021. 10.1007/978-1-4842-6616-8_3

[38] Salunkhe, V. G., Khot, S. M., Desavale, R. G., Yelve, N. P. & Jadhav, P. S. June 7, An Integrated Dimension Theory and Modulation Signal Bispectrum Technique for Analyzing Bearing Fault in Industrial Fibrizer. ASME. ASME J Nondestructive Evaluation. August 2024; 7(3): 031006. (2024). 10.1115/1.4065545

[39] Gangavva, C., Alamelu Mangai, L. & Mohit, B. MFCC based ensemble learning method for multiple fault-diagnosis of roller bearing. *Int. j. inf. Tecnol*. **14** (5), 2741–2751 (2022).

